# The effectiveness of supplemental oxygen during exercise training in patients with chronic obstructive pulmonary disease who show severe exercise-induced desaturation: a protocol for a meta-regression analysis and systematic review

**DOI:** 10.1186/s13643-021-01667-9

**Published:** 2021-04-14

**Authors:** Shohei Kawachi, Shuhei Yamamoto, Kenichi Nishie, Takayoshi Yamaga, Manaka Shibuya, Yasunari Sakai, Keisaku Fujimoto

**Affiliations:** 1 Department of Biomedical Laboratory Science, Graduate School of Medicine, Shinshu University, Matsumoto, Japan; 2 Institute for Biomedical Sciences, Shinshu University, Matsumoto, Japan; 3 Department of Rehabilitation, Shinshu University Hospital, Matsumoto, Japan; 4 The First Department of Internal Medicine, Shinshu University School of Medicine, Matsumoto, Japan; 5 The Department of Respiratory Medicine, Iida Municipal Hospital, Iida, Japan; 6 Department of Occupational Therapy, Health Science University, Minamitsuru-gun, Japan; 7 Department of Rehabilitation, Kitasato University Hospital, Sagamihara, Japan; 8 Department of Rehabilitation, Shinshu University Hospital, Matsumoto, Japan; 9 Department of Clinical Laboratory Sciences, Shinshu University School of Health Sciences, 3-1-1, Asahi, Matsumoto, 390-8621 Japan

**Keywords:** Supplemental oxygen, Desaturation, Hypoxemia, Chronic obstructive pulmonary disease, Exercise training

## Abstract

**Background:**

Supplemental oxygen during exercise training is used to increase the training effect of an exercise program in patients with chronic obstructive pulmonary disease (COPD) who show exercise-induced desaturation. Exercise-induced desaturation is not clearly defined in the guidelines; however, it is generally defined in clinical studies as a decrease in SpO_2_ of more than 4% from rest or a decrease to less than 88% during exercise. Although some meta-analyses examined the effectiveness of supplemental oxygen during exercise training, these studies concluded that it does not further improve exercise tolerance compared to exercise training alone. However, supplemental oxygen during exercise training may be effective in improving exercise tolerance in COPD patients with severe exercise-induced desaturation. Therefore, this study will be performed to elucidate the effectiveness of supplemental oxygen during exercise training and the relationship between its effectiveness and severity of exercise-induced desaturation at baseline.

**Methods:**

We will first assess the effectiveness of supplemental oxygen during exercise training in COPD. The main outcome is the change in exercise tolerance before and after the intervention, indicated by the 6-min walking distance, the walking distance, or the walking time in incremental shuttle walking test, and analyzed as the standardized mean difference (SMD). The quality and risk of bias in individual studies will be assessed using the Grading of Recommendations Assessment, Development, and Evaluation (GRADE) system and risk-of-bias tool (RoB ver.2). If statistical heterogeneity in terms of the effectiveness of exercise tolerance is shown, we will conduct meta-regression analyses to examine the association between the effectiveness of exercise training with supplemental oxygen and severity of exercise-induced desaturation at baseline.

**Discussion:**

One strength of this study is that it is a systematic review with meta-regression analysis to elucidate the effectiveness of supplemental oxygen during exercise training in patients with COPD who show severe exercise-induced desaturation. Furthermore, we will assess the severity of exercise-induced desaturation for which exercise training with supplemental oxygen is effective, the influence of acute effects at baseline, and the effect of supplemental oxygen on adverse events.

**Systematic review registration:**

Registration number, UMIN000039960.

## Description of the condition

Chronic obstructive pulmonary disease (COPD) is a preventable and treatable disease characterized by persistent respiratory symptoms and airflow limitation due to airway and alveolar abnormalities caused by exposure to harmful particles or gasses [1]. An epidemiological study estimated that COPD has an incidence of 384 million in 2020, with a prevalence of 11.7% worldwide [2]. Globally, 3 million people die from COPD annually [3].

As it has been reported that exercise training in rehabilitation improves exercise tolerance, which is related to mortality in COPD [4], the Global Initiative for Chronic Obstructive Lung Disease (GOLD) criteria recommend rehabilitation regardless of the severity of COPD [1]. However, as COPD patients who show exercise-induced desaturation are not able to tolerate high-intensity training, its effectiveness in such cases is limited. In COPD, exercise-induced desaturation is caused by decreases in diffusion capacity and ventilation-perfusion mismatch due to alveolar destruction and airflow obstruction, respectively [5, 6]. Exercise-induced desaturation is not clearly defined in the guidelines; however, it is generally defined in clinical studies as a decrease in SpO_2_ of more than 4% from rest or a decrease to less than 88% during exercise. The patients with severe COPD have more severe exercise-induced desaturation as a result of worsening alveolar hypoventilation by dynamic lung hyperinflation related to airflow limitation on exertion [7, 8]. As exercise-induced desaturation is involved in adverse sequelae and higher risk of mortality in COPD [9, 10], supplemental oxygen during exercise training has been prescribed in COPD patients who show severe desaturation.

The effectiveness of combined supplemental oxygen and exercise training remains unclear. Previous studies showed a twofold increase in exercise training load in the supplemental oxygen group compared to the control group, and therefore, the supplemental oxygen group also showed improvements in exercise tolerance, dyspnea, and depressive symptoms [11, 12]. However, other studies failed to show significant improvements in exercise tolerance with supplemental oxygen [13, 14]. In 2007 and 2019, meta-analyses were conducted to elucidate the effectiveness of supplemental oxygen during exercise training [15, 16]. The meta-analysis in 2019 concluded that supplemental oxygen during exercise training does not further improve exercise tolerance compared to exercise training alone [16]. However, especially in COPD patients with severe exercise-induced desaturation, supplemental oxygen during exercise training may be effective in improving exercise tolerance. In moderate to severe COPD patients with SpO_2_ during exercise ≥ 90%, endurance training three times/week resulted in significant improvements in quality of life (QOL) and exercise capacity independent of oxygen supplementation; however, there were no further benefits of supplemental oxygen from 12 to 24 weeks despite progressively increased training loads [17]. Therefore, we will conduct meta-regression analyses to examine the association between the effectiveness of exercise training with supplemental oxygen and the severity of exercise-induced desaturation. A previous study reported that patients with exercise-induced desaturation and who improved exercise tolerance of 10% or more by ambulatory oxygen on endurance shuttle walk test at baseline showed significant improvements in exercise tolerance by a 6–7-week exercise training program with supplemental oxygen [18]. Therefore, we will also conduct meta-regression analyses to examine the association between the effectiveness of exercise training with supplemental oxygen and acute effects of supplemental oxygen at baseline as a subgroup analysis.

## Objectives

The objective of this systematic review and meta-regression analysis is to elucidate the effectiveness of supplemental oxygen during exercise training and the relationship between its effectiveness and severity of desaturation in COPD.

## Methods and analyses

This systematic review will be conducted according to the Cochrane Handbook for Systematic Review of Interventions, the Preferred Reporting Items for Systematic Reviews, and reported according to the standards of Meta-Analyses (PRISMA) statement [19, 20]. The Grading of Recommendations Assessment, Development, and Evaluation (GRADE) system will be used to assess the evidence in cumulative evidence [21]. This protocol paper was prepared according to PRISMA-P [22].

### Type of study

Randomized controlled trials (RCTs) regardless of cluster or individual randomization until March 31, 2021, will be included restricted to those published in English. Also, full-text studies, those published as abstracts only, and unpublished data are eligible for inclusion. We will only include RCTs with supplemental oxygen during exercise training for COPD and exclude RCTs related to ambulatory oxygen or long-term oxygen therapy.

### Type of participants

We will include participants 18 years of age or older, with a diagnosis of COPD diagnosed according to the GOLD criteria [1]. We will exclude participants with other respiratory diseases including asthma and COPD overlap and combined pulmonary fibrosis and emphysema.

### Type of intervention and comparators

The active treatment group will receive supplemental oxygen during exercise training using wall oxygen units in a hospital or clinic, portable oxygen cylinders, liquid oxygen canisters, and oxygen concentrators.

The control group will be provided sham gas, such as compressed air, through a wall unit or cylinders or will receive no intervention under room air.

The exercise training performed in both groups is defined as a supervised or unsupervised inpatient, outpatient, home-based intervention that includes some form of exercise training applied to COPD patients.

### Types of outcome

#### Primary outcome

1. Exercise capacity, e.g., 6-min walking distance (6MWD: m), shuttle walking distance (SWD: m), the walking time in incremental shuttle walking test (SWT: min), peak oxygen consumption. (Peak VO_2_: mL/kg/min).
2. Maximum exercise load of exercise training, e.g., workload (Watts).
3. Dyspnea scores at the end of the exercise test, e.g., Borg score.
4. Health-related QOL assessed by questionnaires, e.g., 36-Item Short Form Health Survey (SF-36).
5. Number of adverse events, e.g., adverse events assessed by Common Terminology Criteria for Adverse Events (CTCAE)

### Search methods for identification of studies

#### Electronic searches

The meta-analysis should be conducted according to the Cochrane handbook guidance and reported according to PRISMA Statement [19, 20]. We will conduct searches for studies with inception dates up to March 31, 2021, in the Cochrane Central Register of Controlled Trials (CEN-TRAL), MEDLINE, and EMBASE electronic databases. We will also search abstracts and meeting presentations utilizing the same search terms from Medical Subject Headings (MeSH). The search strategy will be based on discussions with an information specialist and will be modified appropriately for each database. The search strategy includes a combination of free text words, words in titles/abstracts, and medical subject headings, such as “pulmonary disease, chronic obstructive,” “supplemental oxygen,” “oxygen inhalation therapy,” and “exercise.” Searches are limited to peer-reviewed research involving human participants. The detailed strategy and details of the formula that will be used to search the databases are shown in Table 1.

**Table 1:** The detailed strategy and details of formula for search in databases

(A). MEDLINE 1. Lung Diseases [MeSH Terms] 2. Lung Diseases, Obstructive [MeSH Terms] 3. Pulmonary Disease, Chronic Obstructive [MeSH Terms] 4. Bronchial Diseases [MeSH Terms] 5. Emphysema [MeSH Terms] 6. Hypoxia [MeSH Terms] 7. Hypoxemia [MeSH Terms] 8. COPD [All Fields] 9. COAD [All Fields] 10. COBD [All Fields] 11. exercise induced-desaturation [Title/Abstract]) 12. #1OR #2 OR #3 OR #4 OR #5 OR #6 OR #7 OR #8 OR #9 OR #10 13. supplemental oxygen [Title/Abstract] 14. oxygen Inhalation Therapy [MeSH Terms] 15. oxygen supplementation [Title/Abstract] 16. oxygen supply [Title/Abstract] 17. Oxygen [MeSH Terms] 18. #13 OR #14 OR #15 OR #16 OR #17 19. exercise [MeSH Terms] 20. resistance Training [MeSH Terms] 21. exercise Therapy [MeSH Terms] 22. high-Intensity Interval Training [MeSH Terms] 23. physical Therapy Modalities [MeSH Terms] 24. physical Conditioning, Human [MeSH Terms] 25. rehabilitation [MeSH Terms] 26. #19 OR #20 OR #21 OR #22 OR #23 OR #24 27. randomized Controlled Trial [Title/Abstract] 28. trial [Title/Abstract] 29. randomized [Title/Abstract] 30. randomized [Title/Abstract] 31. randomly [Title/Abstract]) 32. placebo [Title/Abstract] 33. #27 OR #28 OR #29 OR #30 OR #31 OR #32 34. Animals [Title/Abstract]) 35. #33 NOT #34 36. #12 AND #18 AND #26 AND #35	
(B). Embase 1. lung diseases 2. obstructive lung diseases 3. chronic obstructive pulmonary disease 4. bronchial diseases 5. emphysema 6. hypoxia 7. hypoxemia 8. COPD: ab 9. exercise induced-desaturation 10. #1 OR #2 OR #3 OR #4 OR #5 OR #6 OR #7 OR #8 OR #9 11. oxygen inhalation therapy 12. supplemental oxygen 13. oxygen supplementation 14. oxygen supply 15. #11 OR #12 OR #13 OR #14 16. exercise 17. resistance training 18. exercise therapy 19. interval training 20. physical therapy 21. rehabilitation 22. physical conditioning 23. exercise training 24. #16 OR #17 OR #18 OR #19 OR #20 OR #21 OR #22 OR #23 25. randomized controlled trial 26. trial. ab 27. randomized. ab 28. randomly. ab 29. placebo 30. #25 OR #26 OR #27 OR #28 OR #29 31. animals. ab 32. #30 NOT #31 33. #10 AND #15 AND #24 AND #32	
(C). Cochrane Central Register of Controlled Trials 1. MeSH descriptor: [Lung Diseases] explode all trees 2. MeSH descriptor: [Lung Diseases, Obstructive] explode all trees 3. MeSH descriptor: [Pulmonary Disease, Chronic Obstructive] explode all trees 4. MeSH descriptor: [Bronchial Diseases] explode all trees 5. MeSH descriptor: [Emphysema] explode all trees 6.7MeSH descriptor: [Hypoxia] explode all trees 7. Hypoxemia 8. COPD: ti,ab,kw OR COAD: ti,ab,kw OR COBD: ti,ab,kw OR exercise induced-desaturation: ti,ab,kw 9. #1 OR #2 OR #3 OR #4 OR #5 OR #6 OR #7 OR #8 10. MeSH descriptor: [Oxygen Inhalation Therapy] explode all trees 11. supplemental oxygen 12. oxygen supplementation 13. oxygen supply 14. MeSH descriptor: [Oxygenators] explode all trees 15. #10 OR #11 OR #12 OR #13 OR #14 16. MeSH descriptor: [Exercise] explode all trees 17. MeSH descriptor: [Resistance Training] explode all trees 18. MeSH descriptor: [Exercise Therapy] explode all trees 19. MeSH descriptor: [High-Intensity Interval Training] explode all trees 20. MeSH descriptor: [Physical Therapy Modalities] explode all trees 21. MeSH descriptor: [Physical Conditioning, Human] explode all trees 22. Rehabilitation 23. #16 OR #17 OR #18 OR #19 OR #20 OR #21 OR #22 24. Randomized Controlled Trial: pt OR trial: ti,ab,kw OR randomized: ti,ab,kw OR placebo: ti,ab,kw OR randomly: ti,ab,kw 25. animals 26. #24 NOT #25 27. #9 AND #15 AND #23 AND#26	

#### Searching other resources

We will check the reference lists of all primary studies and review articles for additional references. Further, we will contact experts in the field to ask if they know of any ongoing or unpublished trials.

### Selection of studies

Two review authors (SK and SY) will independently screen the titles and abstracts of all potentially relevant studies identified by the search and code them as “retrieve” (eligible or potentially eligible/unclear) or “do not retrieve.” In the case of disagreements, other review authors will be asked to arbitrate (KN and TY). SK and SY will retrieve the full text reports/publications and independently screen the full texts and identified studies for inclusion. SK and SY will also identify and record reasons for the exclusion of ineligible studies. Any disagreements will be resolved by discussion or by consulting other review authors (KN and TY) when necessary. The review authors will identify and exclude duplicates and collate multiple reports of the same study so that the unit of interest in the review will be each study, rather than each report. We will record the selection process in sufficient detail to complete a PRISMA flow diagram and “Characteristics of excluded studies” table. A data collection form piloted in at least one study in the review will be used to record study characteristics and outcome data. Two review authors (SK and SY) will extract characteristics from the included studies as follows:

1. Methods: study design, total duration of study, duration of follow-up, number of subjects, study setting, and date of study.
2. Participants: sample size, mean age, sex, history of smoking, history of oxygen therapy, forced expiratory volume in 1 s (FEV_1_) after bronchodilator administration, and arterial partial pressure of oxygen (PaO_2_) at baseline.
3. Interventions: type of supplemental oxygen device, oxygen supplemental settings, such as continuous flow or demand delivery systems, and their flow rates, type of intervention as control, such as compressed air (and the flow rate), or room air, type of exercise training as frequency, duration, and contents.
4. Outcomes: exercise capacity, maximum exercise load of exercise training, dyspnea scores at the end of exercise test, health-related QOL assessed by questionnaire survey, number of adverse events assessed by Common Terminology Criteria for Adverse Events (CTCAE).
5. Notes: funding for trial and conflicts of interest of the trial authors.

### Data collection of studies

Two review authors (SK and SY) will independently extract outcome data from the included studies and check each other’s data extraction. One review author (SK) will transfer data into the Review Manager 5.3 file (RevMan 2019) and the EZR statistical program [23]. SK and SY will double-check that data have been entered correctly by comparing the data presented in the systematic review with the study reports. A second review author (KN) will spot-check the study characteristics for accuracy against the trial report.

### Assessment of risk of bias

Two review authors (SK and SY) will independently assess risk of bias for each study using the criteria outlined in the (RoB version 2) in the Cochrane Handbook for Systematic Reviews of Interventions [19]. Any disagreements will be resolved by discussion, by involving other review authors (KN and YT) or by contacting the authors of the included studies. We will assess risk of bias according to the following domains:

1. Bias arising from the randomization process
2. Bias due to deviations from intended interventions
3. Bias due to missing outcome data
4. Bias in measurement of the outcome
5. Bias in selection of the reported result

We will grade each potential source of bias as low risk, some concerns, or high risk and provide a quote from the study report together with a justification for our judgment in the “Risk of bias” table. We will summarize the risk of bias judgments across different studies for each of the domains listed.

### Measures of treatment effect

We will analyze continuous data as the mean difference (MD) or standardized mean difference (SMD) with 95% confidence interval (95% CI). The MD is the absolute difference between the mean change before and after the intervention in a trial. The SMD is used as a summary statistic in the meta-analysis when the studies all assessed the same outcome but measured it in a variety of ways. We will enter data presented as a value with a consistent direction of effect. When the standard deviation (SD) for change from the baseline is not available, we will calculate the SD using Review Manager (RevMan) version 5.3. All CIs have two-sided probability coverage of 95%. A *p* value < 0.05 will be considered significant. We will carry out sensitivity analysis when there is a high risk of bias that could affect the results.

### Assessment of heterogeneity

We will assess statistical heterogeneity in each meta-analysis with Tau^2^, *I*^2^, and *χ*^2^ statistics following the Cochrane Handbook for Systematic Reviews. We regard heterogeneity as substantial with *I*^2^ > 0% and either the Tau^2^ is > zero or *p* < 0.10 in the *χ*^2^ test for heterogeneity. Data from each study will be pooled with fixed-effects modeling when there is no heterogeneity (*p* > 0.1, *I*^2^ ≤ 40%). We will perform meta-analyses with random-effects models when there is heterogeneity (*p* < 0.1, *I*^2^ > 40%).

### Subgroup analysis

We plan to carry out the following subgroup analyses for primary outcomes if we obtain an *I*^2^ score > 50%.

1. Severity of hypoxemia of rest at baseline (PaO_2_ ≤ 60 Torr or PaO_2_ > 60 Torr)
2. Severity of exercise-induced desaturation at baseline (exercise induced desaturation: either decrease of SpO_2_ ≥ 4% and nadir SpO_2_ during exercise ≤ 88% or not-exercise induced desaturation)
3. Severity of airflow limitation (%FEV_1_ ≥ 50% or %FEV_1_ < 50%)
4. Type of outcome (6MWD, SWD, Peak VO_2_, SF-36)
5. Presence or absence of acute effects of supplemental oxygen at baseline (the subjects who improved their exercise tolerance by 10% or more with supplemental oxygen in the exercise load test at baseline or not)
6. Type of exercise training (interval training or not)

We plan to explore differences in outcomes in these subgroups if the number of collected studies is sufficient.

### Sensitivity analysis

We plan to carry out sensitivity analysis for primary outcomes if the high risk of bias of some of the included studies affected the results. We will define “high risk” in each study as at least one domain evaluated as “high risk” or some domains are allocated as “some concern” that can decrease the reliability of the results [19]. We plan to carry out the following sensitivity analyses.

### Meta-regression

If there is any statistically heterogeneity or methodological heterogeneity, we will perform meta-regression using PaO_2_ at baseline (Torr) and decreases of SpO_2_ in exercise tests as covariates to investigate whether the severity of desaturation at baseline affects the effectiveness of exercise tolerance.

### Assessment of reporting biases

When 10 or more studies are included in a meta-analysis, we will create a funnel plot and examine its asymmetry visually to explore any publication bias.

### Assessment of evidence in individual study

We will use the GRADE criteria to assess the quality of evidence for each outcome according to four levels (high, moderate, low, or very low) [21]. The quality of evidence will be reduced by any one of the following limitations: risk of bias, inconsistency, indirectness, imprecision, and publication bias. Two investigators (SK, SY) will independently conduct those assessments. Investigators will resolve disagreements between the two investigators through discussion, with a third reviewer available for adjudication (KN).

## Discussion

The meta-analysis in 2019 concluded that supplemental oxygen during exercise training does not further improve exercise tolerance compared to exercise training alone [16]. However, especially in COPD patients with severe exercise-induced desaturation, supplemental oxygen during exercise training may be effective in improving exercise tolerance. Exercise-induced desaturation is generally defined in clinical studies as a decrease in SpO_2_ of more than 4% from rest or a decrease to less than 88% during exercise [24]; however, it is not clearly defined in the guidelines. Also, it has not been reported for which severity of exercise-induced desaturation supplemental oxygen during exercise training is most effective. Based on the acute physiological response to supplemental oxygen during exercise in patients with moderate to severe COPD [25, 26], it is expected to effective at least COPD patients who have moderate to severe exercise-induced desaturation. Therefore, we will conduct meta-regression analyses to examine the association between the effectiveness of exercise training with supplemental oxygen and the severity of exercise-induced desaturation.

We will conduct several subgroup analyses in this meta-analysis to assess the heterogeneity of the effects of supplemental oxygen during exercise training. In previous studies, as supplemental oxygen during exercise training was used to achieve higher effects associated with higher exercise loads, the setting of supplemental oxygen was determined, but the targets of SpO_2_ were not set [12, 14].Therefore, in this meta-analysis, we will confirm whether the exercise training with supplemental oxygen group achieves a higher exercise load. We will also consider the influence of acute effects in supplemental oxygen therapy at baseline. A previous study reported that patients with exercise-induced desaturation and who improved exercise tolerance of 10% or more by ambulatory oxygen on endurance shuttle walk test at baseline showed significant improvements in exercise tolerance by a 6–7-week exercise training program with supplemental oxygen [18]. Therefore, we will conduct a subgroup analysis to examine the association between the effectiveness of exercise training with supplemental oxygen and acute effects of supplemental oxygen at baseline as a subgroup analysis. The quantity and quality of the trials will possibly be the main limitation of this research. In particular, the meta-analysis of the previous study has reported that there was heterogeneity of effects between types of training, such as interval training or not [15]. We will assess statistical heterogeneity between types of training. The adverse events of supplemental oxygen during exercise training will also be assessed as an outcome. Although previous studies have reported the frequent use of supplemental oxygen during exercise training to avoid desaturation [27], it has not been shown to reduce adverse events [14].

## Data Availability

The datasets generated during and/or analyzed during the current study are available from the corresponding author on reasonable request.

## Abbreviations

COPD: Chronic obstructive pulmonary disease
SMD: Standardized mean difference
GRADE: Grading of Recommendations Assessment, Development, and Evaluation
GOLD: Global initiative for chronic obstructive lung disease
QOL: Quality of life
PRISMA: Preferred Reporting Items for Systematic Reviews, Meta-Analyses
RCT: Randomized controlled trial
6MWD: 6-min walking distance
SWD: Shuttle walking distance
Peak VO_2_: Peak oxygen consumption
SF-36: 36-Item Short Form Health Survey
CEN-TRAL: Cochrane Central Register of Controlled Trials
MeSH: Medical Subject Headings
FEV_1_: Forced expiratory volume in 1 s
PaO_2_: Arterial partial pressure of oxygen
MD: Mean difference
95% CI: 95% confidence interval
